# A Novel Reconstruction Method of K-Distributed Sea Clutter with Spatial–Temporal Correlation

**DOI:** 10.3390/s20082377

**Published:** 2020-04-22

**Authors:** Mingyue Ding, Yachao Li, Yinghui Quan, Liang Guo, Mengdao Xing

**Affiliations:** 1 National Laboratory of Radar Signal Processing, Xidian University, Xi’an 710071, China; myding@stu.xidian.edu.cn (M.D.); yhquan@mail.xidian.edu.cn (Y.Q.); xmd@xidian.edu.cn (M.X.); 2 The School of Physics and Optoelectronic Engineering, Xidian University, Xi’an 710071, China; lguo@mail.xidian.edu.cn

**Keywords:** spatial–temporal correlated sea clutter, proportional method, compound model, K-distribution

## Abstract

The reconstruction of sea clutter plays an important role in target detection and recognition in a maritime environment. Reproducing the temporal and spatial correlations of real data simultaneously is always a problem in the reconstruction of sea clutter due to the complex coupling between them. In this paper, the spatial–temporal correlated proportional method (STCPM), based on a compound model, is proposed to reconstruct K-distributed sea clutter with correlation characteristics obtained from the real data. The texture component with spatial–temporal correlation is generated by the proportional method and the speckle component with temporal correlation is generated by matrix transformation. Compared with previous methods, the biggest innovation of the STCPM is that it can more accurately generate K-distributed sea clutter with both temporal and spatial correlations. The comparison of the reconstructed and real data demonstrates that the method can reproduce the characteristics of real sea clutter well.

## 1. Introduction

Sea clutter is one of the most important restricting factors for target detection and recognition in a maritime environment. The design and selection of radars and radar signal processing algorithms for use in a sea clutter environment is directly influenced by knowledge of sea clutter characteristics. The accurate reconstruction of sea clutter can provide a firm foundation for the research of targets detection and recognition algorithms in a maritime environment.

There are abundant factors that determine the characteristics of the sea clutter, including the sea state, the wind speed, duration and direction, the wave speed, the fetch, and the swell direction [1], which can account for the extremely complex physical mechanism of sea clutter. In addition, the continuous and complex changes of the above natural factors lead to the instability of the sea clutter, which makes the modeling and reconstruction of sea clutter a challenge.

It is necessary to study and analyze the characteristics of the sea clutter deeply before the accurate reconstruction of sea clutter. For past few decades, many efforts have been devoted to the research of sea clutter characteristics, including the amplitude distribution [2,3,4,5,6], correlation characteristics [7,8,9,10,11], Doppler spectrum [12,13,14,15,16], spikes [17,18,19,20], non-stationarity [21,22], and so on. Among them, amplitude distribution and correlation characteristics get the most attention, and they play an important role in the design of target detection algorithms in a maritime environment. 

Referring to amplitude distribution models, the K-distribution [23,24,25] is one of the most widely used distribution models. The K-distribution is a compound model, which not only fits with the probability distribution of sea clutter intensities in many cases, but corresponds well to the scattering mechanism of sea clutter. For a modern radar system with a fine resolution and a high pulse repetition frequency (PRF), the received sea clutter returns are spatial–temporal correlated. The spatial correlation of sea clutter is defined as the cross-correlation between the signals returned from two separate patches of the sea in the radial dimension, and it has great significance to the target detection, which provides a basis for selecting the reference bins. The temporal correlation of the sea clutter is defined as the correlation of returns from different pulses in a single range bin. The temporal correlation and Doppler spectra, relevant to the former via the Fourier transform, are usually used to design radar optimal waveform. The accurate modeling of the amplitude and correlation characteristics of real sea clutter is the basic requirement of sea clutter reconstruction and subsequent signal processing in radar system.

Many studies were conducted on the reconstruction of spatially or temporally correlated sea clutter, but there were few methods for the reconstruction of spatial–temporal correlated sea clutter. The authors of [26,27] gave the theoretical derivation of spatial correlation models and achieved the reconstruction of spatially correlated sea clutter barely. In [28], spatial–temporal correlated sea clutter real sequences of three amplitude distributions were generated, but there is no phase information of real data. A relatively complete method of reconstructing spatial–temporal correlated K-distributed sea clutter was proposed in [1], where the spatial and temporal correlations were individually controlled, respectively. In [29], two methods of simulating temporally correlated sea clutter in short term and long term were discussed separately. The spatial and temporal correlations of clutter were considered to be independent there; however, the texture component of clutter is spatial–temporal correlated in the long-term and there is complex coupling between the spatial and temporal correlations. Because the simultaneously control of spatial and temporal correlations of texture is difficult, the correlation characteristics of real sea clutter cannot be reproduced well in many reconstruction algorithms.

The spherically invariant random process (SIRP) method [30,31,32] and the zero-memory nonlinearity (ZMNL) method [32,33,34] are commonly used methods in sea clutter reconstruction. The ZMNL method is suitable for the reconstruction of clutter with integral shape parameter, but it often produces errors for non-integers or semi-integers. In addition, the sea clutter sequences generated by ZMNL method have no phase information. In the SIRP method, the sea clutter is considered as an SIRP, indeed, the method is applicable only if sea clutter is observed and processed on time intervals much shorter than the average decorrelation time of the modulating component. 

In this paper, a novel spatial–temporal correlated proportional method (STCPM) based on a compound model for the reconstruction of K-distributed sea clutter with spatial–temporal correlation is proposed. The method extends the idea introduced in [1], where only the temporal correlation of speckle and spatial correlation of texture are presented based on the compound K-distribution model for clutter amplitude statistics. Compared with the existing algorithms, the biggest innovation of STCPM is that it can generate the texture component with both temporal and spatial correlations. In addition, the cross-correlation between the real and imaginary parts of the speckle and the coherence time estimation of texture are also taken into account in the STCPM, which reproduces the correlation characteristics of real sea clutter. The comparison of the reconstructed and real data shows that the proposed method can describe the sea clutter more accurately in amplitude and correlation characteristics. 

The remainder of this paper is organized as follows. Section 2 makes a brief description of the characteristics and traditional reconstruction method of sea clutter. Section 3 completes the analysis of real data. Section 4 focuses on the reconstruction of spatial–temporal correlated sea clutter returns. Section 5 presents the reconstruction results with detailed analysis. The last section concludes this paper.

## 2. Characteristics and Reconstruction Method of Sea Clutter

### 2.1. Amplitude and Correlation Characteristics

#### 2.1.1. K-Distribution

The K-distribution is a compound Gaussian model with two main components to model the fluctuations of sea clutter. The first component is the local mean level y obeying a generalized Chi-distribution, thus there is a gamma-distributed local clutter power (texture). The second component termed the “speckle”, a complex Gaussian process with a Rayleigh-distributed envelope, which is modulated by the first component. The probability density function (PDF) of overall amplitude of the sea clutter is given by [35]

(1) $$f\left(x\right)=\int_{0}^{\infty }f\left(x|y\right)f\left(y\right)dy,\begin{array}{cc} & 0\leq x\leq \infty \end{array}$$

where

(2) $$f\left(x|y\right)=\frac{\pi x}{2y^{2}}\exp\left(-\frac{\pi x^{2}}{4y^{2}}\right)$$

(3) $$f\left(y\right)=\frac{2b^{2v}y^{2v-1}}{\Gamma \left(v\right)}\exp\left(-b^{2}y^{2}\right),\begin{array}{cc} & 0\leq y\leq \infty \end{array}$$

where Γ(v) is the gamma function; v and b are the shape and scale parameters of gamma distribution, respectively.

#### 2.1.2. Correlation

The temporal correlation of sea clutter is often influenced by the correlation characteristics of the speckle and the texture components. The speckle component from any individual range bin has a short temporal decorrelation period (typically a few milliseconds). In contrast, the texture component is not affected by frequency agility, and it has a long temporal decorrelation period on the order of seconds. As a result, the speckle component is called a fast fluctuating component, whereas the texture component is called a slowly fluctuating component.

Usually, the small-scale features at two spatially separated patches are uncorrelated [1], and the spatial correlation of sea clutter shows a similar trend with that of the large-scale features. Thus, the speckle is usually assumed to be uncorrelated between different range bins and the overall spatial correlation of sea clutter is mainly dependent on the modulating component (texture). 

The correlation coefficient can be calculated as follows,

(4) $$\rho_{x}\left(k\right)=\frac{\sum_{n=1}^{N/2}\left(x\left[n\right]-\bar{x}\right)\left(x^{*}\left[n+k\right]-\bar{x^{*}}\right)}{\sqrt{\sum_{n=1}^{N/2}{\left|\left(x\left[n\right]-\bar{x}\right)\right|}^{2}}\sqrt{\sum_{n=1}^{N/2}{\left|\left(x^{*}\left[n+k\right]-\bar{x^{*}}\right)\right|}^{2}}}$$

where x[n] is the complex received signal, $x^{*}$ and $\bar{x}$ denote the complex conjugate and the average of x, respectively.

#### 2.1.3. Coherence

According to the K-distribution model, the discrete-time expression of received complex clutter samples z[n] can be written as follows [36],

(5) $$z\left[n\right]=\sqrt{\tau \left[n\right]}g\left[n\right]$$

where τ[n] and g[n] represent the texture and speckle components, respectively.

As the correlation time of texture is much longer than that of the speckle, the received clutter sequence can be regarded as the product of a compound Gaussian process times a random constant in a short period (the coherence time of the texture) [36], which can be expressed as

(6) $$z\left[n\right]≃\sqrt{\tau \left[k\right]}g\left[n\right],n=k-L_{c}+1,\ldots ,k+L_{c}$$

The texture represents the local mean clutter power of sea clutter, thus the work in [37] gives the estimate of the texture sequence:

(7) $$\hat{\tau }\left[n\right]=\frac{1}{L_{c}}\sum_{k=n-Lc/2}^{n+L_{c}/2-1}{\left|z\left[k\right]\right|}^{2}$$

where $L_{c}$ is the coherence length of the texture component and $\hat{\tau }\left[n\right]$ is the estimate of the texture component. As can be seen, the determination of coherence length has a big significance to the separation of texture from the received clutter data and the analysis of correlation characteristics.

### 2.2. Traditional Method

**Figure 1:**
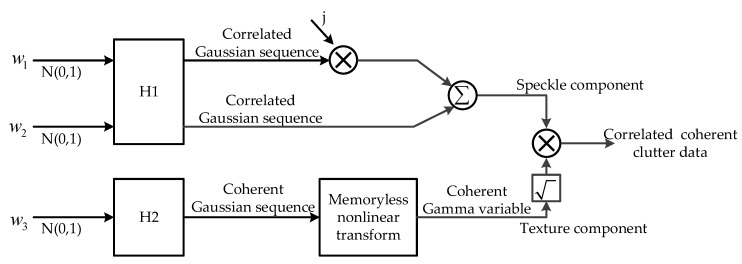
Block diagram of traditional method for generation of coherent and correlated K-distributed clutter.

In a K distribution, the clutter can be modeled as a zero-mean complex correlated Gaussian sequence, modulated by a real, non-negative non-Gaussian variable. The block diagram of traditional method for generation of coherent and temporally correlated K-distributed clutter is shown in Figure 1.

The block diagram contains two branches, where the upper branch is used to generate the speckle component, a complex correlated Gaussian sequence, and the lower branch is used to generate the texture component, a Gamma variable. The three input sequences are all white zero-mean Gaussian sequences with identity variance and independent of each other. The filters H1 and H2 are, respectively, used to generate the temporal correlation of the speckle and coherence of the texture.

In the model, the real and imaginary parts of the speckle component are considered to be mutually independent and the texture component is assumed to be a constant. In practice, there is cross-correlation between the real and imaginary parts of the speckle component, and the texture is not a constant when the reconstruction time exceeds the coherence length, but a correlated Gamma-distributed sequence. In addition, the spatial correlation of clutter is not considered in the method. As a result, the above traditional reconstruction method cannot reproduce the correlation characteristics of sea clutter completely.

## 3. Data Analysis

The ideas presented in this paper are based on the analysis of real data collected on 12 September 2013 at Huludao of Liaoning province, China, using a coherent millimeter-wave radar system. For this trial, the radar was set up on a cliff-top approximately 230 m above the sea surface and was operated in the spotlight mode. The main characteristic parameters of radar are listed in Table 1. 

**Figure 2:**
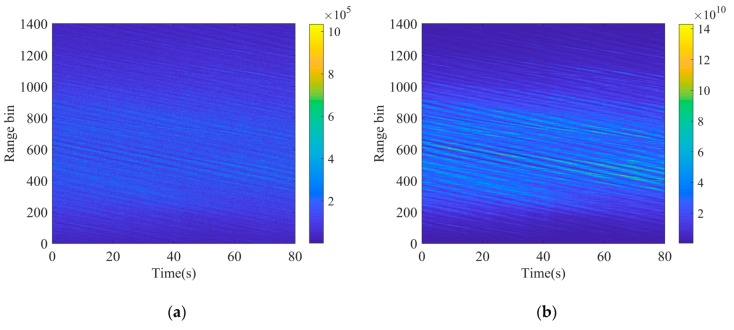
The amplitude-range-time of real data: (**a**) Original clutter; (**b**) Original texture separated from clutter.

The collected data contains 4000 range bins, many of which contain few clutter signals due to the limitation of the antenna beam width. Thus, only the middle 1400 range bins are considered in this paper. Each range bin record comprises complex time-series data recorded over intervals of 80 s, sampled at the PRF. The recording of each range bin contains 40,000 pulse samples (80 s) in this paper. The amplitude-range-time of clutter is shown as Figure 2a.

**Figure 3:**
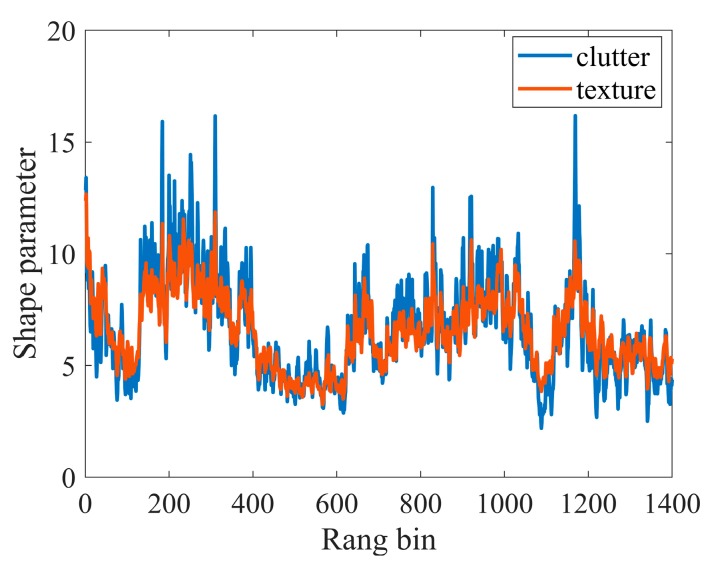
Shape parameters of real clutter and texture in different range bins.

According to the K distribution, the analysis of the real data characteristics is based on the correct separation of speckle and texture components, which can be achieved by the method in [36]. The coherence length of experimental texture is ~200 samples, corresponding to 400ms. Consequently, the texture and speckle components can be separated from the clutter. The amplitude-range-time of texture is shown in Figure 2b. It can be seen that the oblique strips are very similar to the clutter amplitude and there is a square relationship between their amplitude, which can be explained by Equation (7). To further verify the correctness of the separation and accurately generate texture component according to the characteristics of the real data, the shape parameters of real clutter and texture are estimated by the method of Moments (MoM) [38,39,40], which is presented in Figure 3.

Theoretically, the shape parameter of K distribution denoting the clutter amplitude is the same as that of the gamma distribution denoting the texture component. Thus, the great similarity between the clutter and texture in Figure 3 can also show that the above separation of the two components is comparatively accurate.

As shown in Figure 2, the clutter amplitude in middle range bins is much stronger than the marginal range bins. Therefore, the middle 400 range bins, corresponding to the range bins from 401 to 800 in the figure, are chosen to be analyzed in the paper.

### 3.1. Amplitude

**Figure 4:**
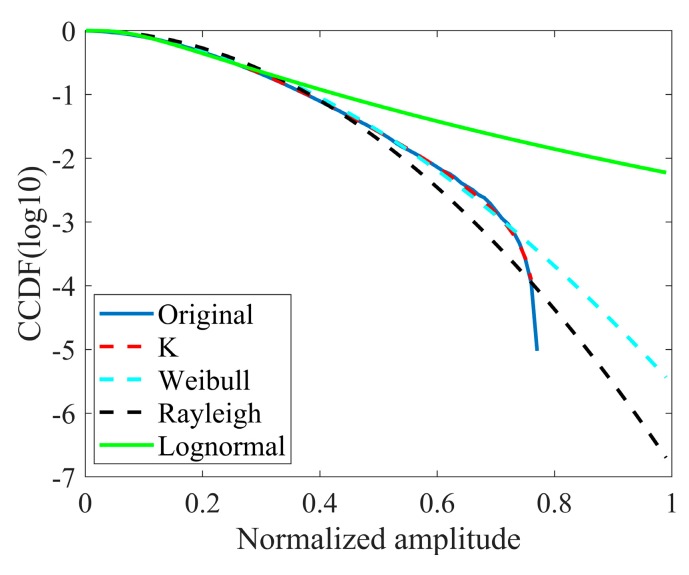
The fitting results of original clutter amplitude (range bin = 300, CNR = 18.97dB).

In order to justify the applicability of compound-Gaussian amplitude models, first-order statistical analysis of the real data is performed here. The models under consideration are the Rayleigh, Weibull, log-normal, and K distributions, where the log-normal distribution is only used to provide a comparison although it is not a compound-Gaussian model. The amplitude fitting results and estimated model parameters are given in Figure 4 and Table 2, respectively.

The parameters in the table, $v_{-}$ and $c_{-}$, represent the shape and scale parameters, respectively. The fitting results show that the empirical PDF is most consistent with the K distribution.

### 3.2. Correlation

#### 3.2.1. The Texture Component

The temporal correlation of the slowly varying component usually requires the participation of a much larger number of samples than that of the fast fluctuating component. As the texture component is approximately constant in the coherence length, that is, the correlation is approximately 1, the temporal correlation of the texture can be estimated as follows.

Assume τ[m,n](m=1,2,…,M;n=1,2,…,N) is the texture data block, M and N stand for the numbers of the range bins and pulses, respectively. A new data block $\tau_{1}\left[m,l\right]\left(m=1,2,\ldots ,M;l=1,2,\ldots ,N/L_{c}\right)$ is constructed by $\left[N/L_{c}\right]$ elements from original data in each range bin, such as $\tau \left[m,1\right],\tau \left[m,1+L_{c}\right],\ldots ,\tau \left[m,1+\left(\left[N/L_{c}\right]-1\right)*L_{c}\right]\left(m=1,2,\ldots ,M\right)$. Therefore, the temporal correlation coefficient is expressed as

(8) $$\rho_{t\tau }\left(k\right)=\frac{\sum_{l=1}^{\left[N/L_{c}\right]/2}\left(\tau_{1}\left[m,l\right]-\bar{\tau_{1}\left(m\right)}\right)\left(\tau_{1}\left[m,l+k\right]-\bar{\tau_{1}\left(m\right)}\right)}{\sqrt{\sum_{l=1}^{\left[N/L_{c}\right]/2}{\left|\left(\tau_{1}\left[m,l\right]-\bar{\tau_{1}\left(m\right)}\right)\right|}^{2}}\sqrt{\sum_{l=1}^{\left[N/L_{c}\right]/2}{\left|\left(\tau_{1}\left[m,l+k\right]-\bar{\tau_{1}\left(m\right)}\right)\right|}^{2}}}$$

where $\bar{\tau_{1}\left(m\right)}=\frac{1}{\left[N/L_{c}\right]}\sum_{l=1}^{\left[N/L_{c}\right]}\tau_{1}\left[m,l\right]$, [·] denotes the complex conjugate.

**Figure 5:**
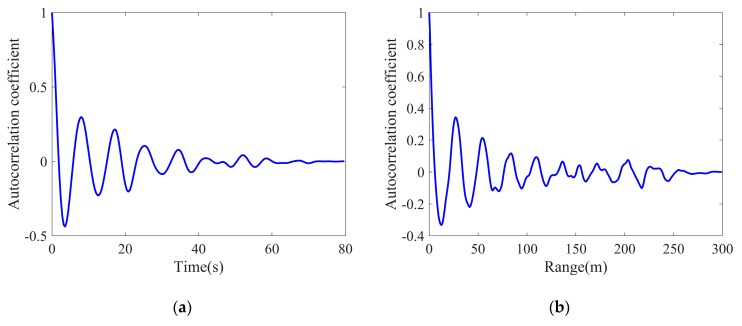
Average correlation coefficients of real texture: (**a**) Temporal correlation; (**b**) Spatial correlation.

The spatial correlation of the texture can be directly calculated from the original sequence, which is expressed as

(9) $$\rho_{s\tau }\left(k\right)=\frac{\sum_{m=1}^{M/2}\left(\tau_{1}\left[m,l\right]-\bar{\tau_{1}\left(l\right)}\right)\left(\tau_{1}\left[m+k,l\right]-\bar{\tau_{1}\left(l\right)}\right)}{\sqrt{\sum_{m=1}^{M/2}{\left|\left(\tau_{1}\left[m,l\right]-\bar{\tau_{1}\left(l\right)}\right)\right|}^{2}}\sqrt{\sum_{m=1}^{M/2}{\left|\left(\tau_{1}\left[m+k,l\right]-\bar{\tau_{1}\left(l\right)}\right)\right|}^{2}}}$$

where $\bar{\tau_{1}\left(l\right)}=\frac{1}{M}\sum_{m=1}^{M}\tau_{1}\left[m,l\right]$. The results are shown in Figure 5; it is obvious that the temporal correlation starts from one and slowly decays in a periodic manner. Similar to the temporal correlation, the spatial correlation of the texture component also performs a periodic attenuation, which corresponds to the fluctuation characteristics of the sea surface.

#### 3.2.2. The Speckle Component

**Figure 6:**
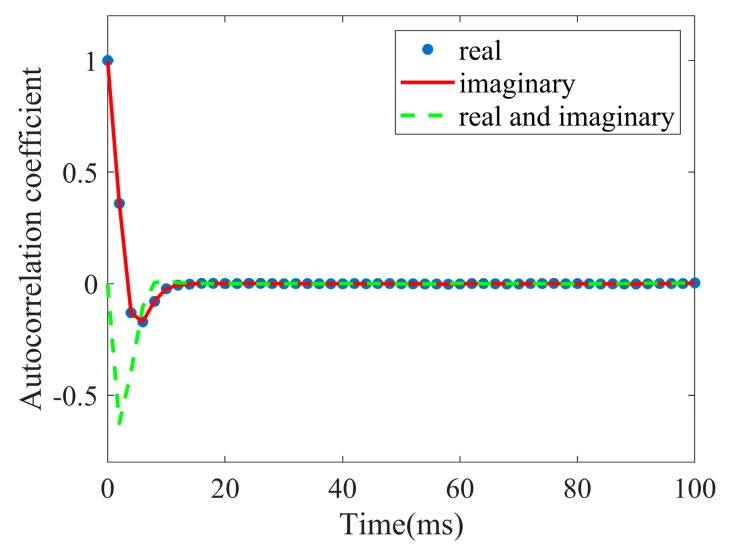
Autocorrelation and cross-correlation coefficients of real and imaginary parts of the speckle component.

The speckle component is a complex Gaussian process; thus, the temporal correlation of speckle includes three parts: respective autocorrelation of the real and imaginary part, and the cross-correlation between the two parts. The three correlation coefficients of speckle components can be estimated according to Equation (4). As shown in the Figure 6, the autocorrelation of the real part is same as that of the imaginary part, which starts from one with an initial rapid decrease and then goes to zero, whereas the cross-correlation starts from zero and shows a negative peak. As can be seen, the autocorrelation time and the cross-correlation time are all within 10ms, which is much shorter than the decorrelation time of the texture.

#### 3.2.3. The Clutter Amplitude

**Figure 7:**
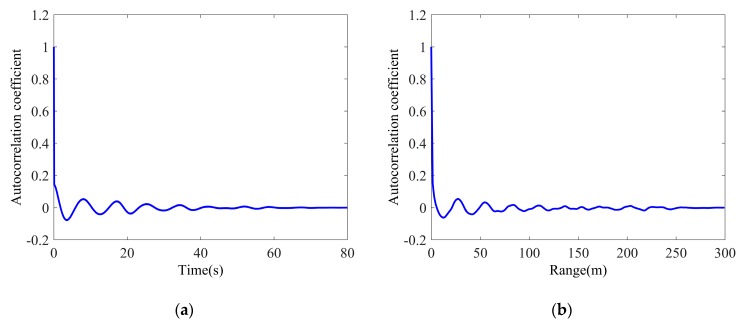
Average correlation coefficients of clutter amplitude: (**a**) Temporal correlation; (**b**) Spatial correlation.

The temporal and spatial correlation coefficients of the clutter amplitude are also estimated by Equation (4). As shown in Figure 7, the temporal correlation coefficient rapidly decreases at first, corresponding to the temporal correlation of the speckle, and then it slowly decays in a periodic manner, which is quite similar to the temporal correlation of the texture. It shows that the short-time correlation characteristics of clutter amplitude are dominated by the speckle component as the texture varies little, whereas the long-time correlation with periodic characteristic is mainly influenced by the texture component. As expected, the periodic manner is also shown in the spatial correlation due to the texture. However, although the speckle samples are assumed to be independent in the different range bins, the spatial correlation still performs a rapid decrease similar to the temporal correlation of clutter amplitude, which indicates there is slight spatial correlation in speckle, a possible reason for this is that the sampling interval is less than the range resolution bin.

## 4. Reconstruction of Spatial–Temporal Correlated Sea Clutter

**Figure 8:**
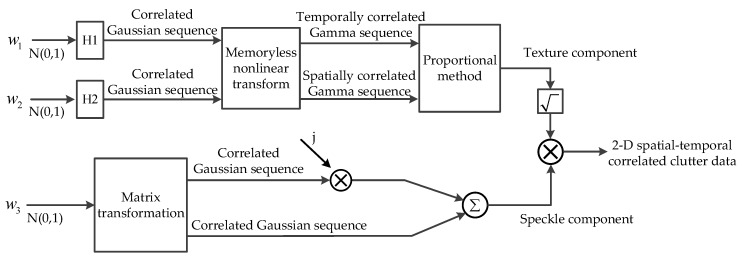
Block diagram of spatial–temporal correlated proportional method (STCPM) for generation of K-distributed clutter with spatial–temporal correlation.

The section describes the proposed method for modeling 2D sea clutter data, based on the observations of real data. The value of any model must be assessed by its ability to reproduce characteristics of the real data. In the paper, a new method, STCPM, based on a compound model of reconstructing K-distributed sea clutter, is proposed, which reproduces both the temporal and spatial correlations of the real sea clutter, including the autocorrelation and cross-correlation of the speckle component, and coherence and spatial–temporal correlation of the texture component. The block diagram of STCPM for generation of K-distributed clutter with spatial–temporal correlation is shown in Figure 8.

Similar to the traditional method, the input of STCPM is also white zero-mean Gaussian sequences, whereas the H1 and H2 are used to generate the temporal and spatial correlations of the texture. The H1 and H2 can be achieved by matrix transformation in the lower branch, which is described in Section 4.2. The detailed reconstruction process is described below.

### 4.1. Reconstruction of the Texture Component 

The 2D spatial–temporal correlated texture can be considered as a block obtained by the fluctuation of a temporally correlated sequence over range. In general, the larger the variation of the sequence in the different range bins is, the smaller the correlation is, and vice versa, which indicates a close connection between the fluctuations in different range bins and the spatial correlation of the texture. Consequently, the texture component can be considered as a process where a temporally correlated sequence is modulated by a spatially correlated sequence. Based on the modulation theory, the proportional method is proposed, where the 2D correlated texture block can be obtained by extending an initial temporally correlated sequence and another initial spatially correlated sequence. This method is based on the assumption that the temporal correlation in the processing interval and spatial correlation within the range bins are unchanged.

**Figure 9:**
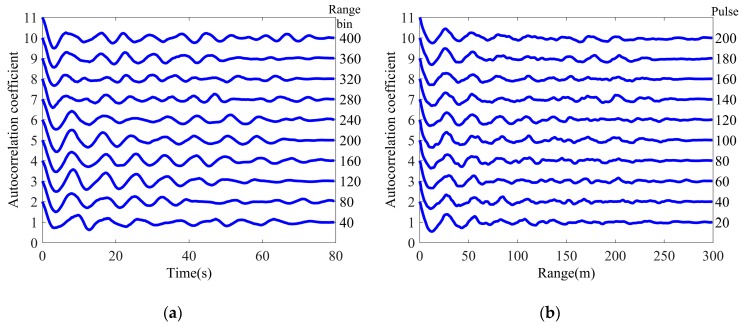
Correlation coefficients in a single range bin and pulse of texture: (**a**) Temporal correlation; (**b**) Spatial correlation.

As shown in Figure 9, the correlation coefficients in different range bins and pulses of the chosen area change a little, thus the above assumption is reasonable. In the paper, the average temporal correlation and the average spatial correlation are used to generate the initial temporally correlated and spatially correlated sequences in the reconstruction of sea clutter. At the same time, the variation of shape parameter is not considered in the paper for the sake of balancing the temporal and spatial correlations. Similar to the correlation, the average shape parameters in range dimension and pulse dimension are used in the reconstruction of sea clutter. The scale parameter is mainly influenced by the amplitude of the signal, and sequences with different scale parameters can be obtained by re-scaling [41], i.e., if a variable Z∼Γ(v;1), then θZ~Γ(v,θ), so the normalized scale parameter is used in the paper.

Attempting to maintain the invariability of the correlations is a significant feature of the proportional method. Indeed, the correlations of the two proportional sequences are identical. As mentioned earlier, the correlation coefficient of a correlated sequence $x=\left\{x_{n}\right\},n=1,2,\ldots ,N$ can be estimated by Equation (4). Similarly, the correlation coefficient of the new sequence $y=\left\{y_{n}\right\}=\left\{\alpha x_{n}\right\},n=1,2,\ldots N$ can be expressed as follows,

(10) $$\begin{array}{ll} \rho_{y}\left(k\right) & =\frac{\sum_{n=1}^{N/2}\left(y\left[n\right]-\bar{y}\right)\left(y^{*}\left[n+k\right]-\bar{y*}\right)}{\sqrt{\sum_{n=1}^{N/2}{\left|\left(y\left[n\right]-\bar{y}\right)\right|}^{2}}\sqrt{\sum_{n=1}^{N/2}{\left|\left(y^{*}\left[n+k\right]-\bar{y^{*}}\right)\right|}^{2}}}\\ & =\frac{\sum_{n=1}^{N/2}\left(\alpha x\left[n\right]-\alpha \bar{x}\right)\left(\alpha x^{*}\left[n+k\right]-\alpha \bar{x^{*}}\right)}{\sqrt{\sum_{n=1}^{N/2}{\left|\left(\alpha x\left[n\right]-\alpha \bar{x}\right)\right|}^{2}}\sqrt{\sum_{n=1}^{N/2}{\left|\left(\alpha x^{*}\left[n+k\right]-\alpha \bar{x^{*}}\right)\right|}^{2}}}\\ & =\frac{\alpha^{2}\sum_{n=1}^{N/2}\left(x\left[n\right]-\bar{x}\right)\left(x^{*}\left[n+k\right]-\bar{x^{*}}\right)}{\alpha \sqrt{\sum_{n=1}^{N/2}{\left|\left(x\left[n\right]-\bar{x}\right)\right|}^{2}}\alpha \sqrt{\sum_{n=1}^{N/2}{\left|\left(x^{*}\left[n+k\right]-\bar{x^{*}}\right)\right|}^{2}}}\\ & =\frac{\sum_{n=1}^{N/2}\left(x\left[n\right]-\bar{x}\right)\left(x^{*}\left[n+k\right]-\bar{x^{*}}\right)}{\sqrt{\sum_{n=1}^{N/2}{\left|\left(x\left[n\right]-\bar{x}\right)\right|}^{2}}\sqrt{\sum_{n=1}^{N/2}{\left|\left(x^{*}\left[n+k\right]-\bar{x^{*}}\right)\right|}^{2}}}\\ & =\rho_{x}\left(k\right) \end{array}$$

where α is proportional parameter and it is a constant.

From the above analysis, it can be seen that there is not any change in correlation and shape parameter of a sequence when its elements change in proportion. As a result, the characteristics of the gamma sequence are used to generate 2D texture data in the proposed proportional reconstruction method. 

Suppose the texture block to be generated is T and its size is M×N, where M and N are the numbers of range bins and pulses, respectively. The specific steps of generating T are summarized as follows.

1. Generate an initial temporally correlated gamma sequence $\left[\tau_{11},\tau_{12},\ldots ,\tau_{1N}\right]$ as the first row of the T;
2. Generate an initial spatially correlated gamma sequence $\left[\tau_{11}^{\prime },\tau_{21}^{\prime },\ldots ,\tau_{M1}^{\prime }\right]$, let $\frac{\tau_{11}^{\prime }}{\tau_{11}}=\frac{\tau_{21}^{\prime }}{\tau_{21}}=\ldots =\frac{\tau_{M1}^{\prime }}{\tau_{M1}}$, then $\tau_{21}=\frac{\tau_{11}}{\tau_{11}^{\prime }}\tau_{21}^{\prime }$, $\tau_{31}=\frac{\tau_{11}}{\tau_{11}^{\prime }}\tau_{31}^{\prime }$,…, $\tau_{M1}=\frac{\tau_{11}}{\tau_{11}^{\prime }}\tau_{M1}^{\prime }$, so the first column of the T can be generated, that is $\left[\tau_{11},\tau_{21},\ldots ,\tau_{M1}\right]$;
3. Let $\frac{\tau_{21}}{\tau_{11}}=\frac{\tau_{22}}{\tau_{12}}=\ldots =\frac{\tau_{2N}}{\tau_{1N}}$, then $\tau_{22}=\frac{\tau_{21}}{\tau_{11}}\tau_{12}$, $\tau_{23}=\frac{\tau_{21}}{\tau_{11}}\tau_{13}$, …, $\tau_{2N}=\frac{\tau_{21}}{\tau_{11}}\tau_{1N}$, so the second row of the T is generated: $\left[\tau_{21},\tau_{22},\ldots ,\tau_{2N}\right]$;
4. Similarly, let $\frac{\tau_{m1}}{\tau_{11}}=\frac{\tau_{m2}}{\tau_{12}}=\ldots =\frac{\tau_{mN}}{\tau_{1N}}$, then $\tau_{m2}=\frac{\tau_{m1}}{\tau_{11}}\tau_{12}$, $\tau_{m3}=\frac{\tau_{m1}}{\tau_{11}}\tau_{13}$, ..., $\tau_{mN}=\frac{\tau_{m1}}{\tau_{11}}\tau_{1N}$, the other rows of T can be obtained in turn.

Therefore, the responding 2D texture data can be generated through the above steps as long as an initial temporally correlated gamma sequence and an initial spatially correlated gamma sequence are generated. 

In the method, the gamma sequence with specified correlation can be generated by the matrix transformation method in Section 4.2 and memoryless nonlinear transform (MNLT). The MNLT is a fairly straightforward way to transform a set of random Gaussian distributed variables to non-Gaussian distributed variables [6]. Suppose the input Gaussian process has a known PDF $f_{1}\left(u\right)$, and the output process has a desired PDF $f_{2}\left(v\right)$, the mapping between the input and out processes is expressed as follows [41],

(11) $$\begin{array}{ll} \int_{v}^{+\infty }f_{2}\left(v^{\prime }\right)dv^{\prime } & =\int_{u}^{+\infty }f_{1}\left(u^{\prime }\right)du^{\prime }\\ & =\frac{1}{\sqrt{2\pi }}\int_{u}^{+\infty }\exp\left(-\frac{u^{\prime }{}^{2}}{2}\right)du^{\prime }\\ & =\frac{1}{2}erfc\left(\frac{u}{\sqrt{2}}\right) \end{array}$$

where erfc(·) is the complementary error function. The complementary quantile function $Q_{dist}\left(\varsigma \right)$ of the required distribution is now defined by

(12) $$\int_{Q_{dist}\left(\varsigma \right)}^{\infty }f_{2}\left(v\right)dv=\varsigma .$$

Thus, the MNLT that takes the input Gaussian random variables into the corresponding values of the required non-Gaussian random variables can be written as follows [23],

(13) $$v=Q_{dist}\left(erfc\left(u/\sqrt{2}\right)/2\right)$$

### 4.2. Reconstruction of the Speckle Component 

Compared with the texture component, the generation of the speckle component is simpler. In some papers [28,42], two independent correlated Gaussian processes are usually taken as the real and imaginary parts of the speckle. However, from the analysis of the real sea clutter data, we know that there is a correlation between the real and imaginary parts of the speckle. In order to simulate the speckle more accurately, the matrix transformation method in [1] is used in the paper, which takes the cross-correlation of the speckle into consideration. The steps are summarized as follows.

1. Generate a 2*N*-dimensional white zero-mean Gaussian random vector $W=\left[w_{i1},\ldots ,w_{iN},w_{q1},\ldots ,w_{qN}\right]$ with the identity covariance matrix;
2. Determine the covariance matrix $M=\left[\begin{array}{cc} M_{ii} & M_{iq}\\ M_{qi} & M_{qq} \end{array}\right]$, where $M_{ii}$ and $M_{qq}$ are the covariance matrices of the real and imaginary components, respectively, $M_{iq}$ and $M_{qi}$ are their cross-covariance matrices;
3. Calculate the transformation matrix $G=ED^{1/2}$, where D is the diagonal matrix of eigenvalues of M and E is the matrix of normalized eigenvectors;
4. Get the correlated Gaussian sequence $V=GW=\left[\begin{array}{cc} V_{i} & V_{q} \end{array}\right]$, where $V_{i}$ and $V_{q}$ are the real and imaginary parts of the speckle, respectively.

The complex sea clutter sequence can be obtained according to the block diagram in Figure 8. In the end, the noise is introduced into the clutter data by the method described in [1].

## 5. Simulation Results

The methods presented in Section 4 can be used to generate 2D K-distributed sea clutter data with correlation characteristics obtained from the real data, which include the temporal correlation, spatial correlation and coherence. The simulated data set consists of 5000 consecutive pulses sampled at 400 range bins, totaling 2,000,000 sample points. In order to offset the randomness of the Gaussian sequences generation in the simulation, the average results presented there is obtained from 1000 trials. At the same time, the classic methods in [1,25] are provided in this paper for comparison, which are used to generate the spatially correlated and temporally correlated texture, respectively. In the following analysis, the contrastive methods are called the spatially correlated method (SCM) and the temporally correlated method (TCM).

**Figure 10:**
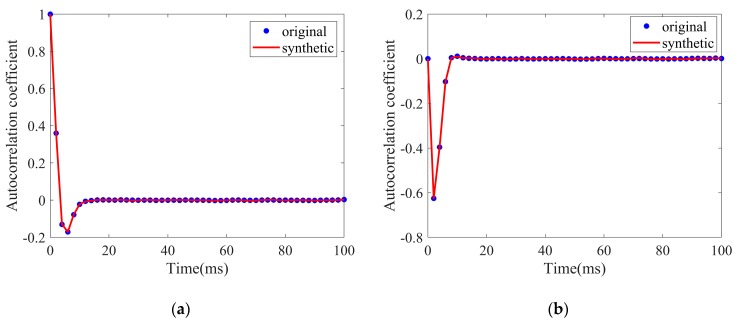
Average correlation coefficients of speckle: (**a**) Autocorrelation; (**b**) Cross-correlation.

Figure 10 presents the comparison of the average autocorrelation and cross-correlation between the original speckle and synthetic speckle. It is clear that the correlation characteristic of the synthetic speckle is very similar to that of the original data.

**Figure 11:**
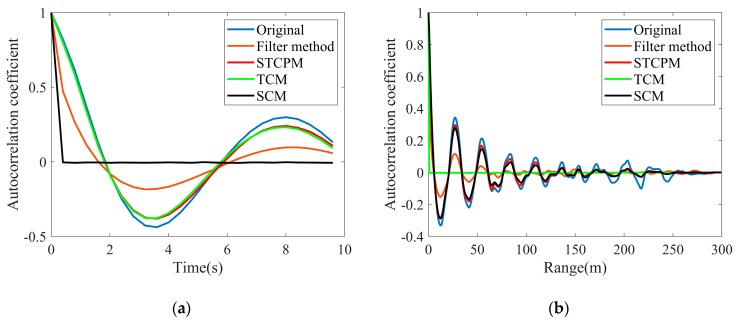
Average correlation coefficients of texture: (**a**) Temporal correlation; (**b**) Spatial correlation.

Figure 11 provides the comparison of the average temporal and spatial correlation coefficients between the original and synthetic textures. In addition to the above methods, a filter method of generating a 2D correlated texture block in [6] is also presented for comprehensive comparison. Figure 11a shows a similarity of the temporal correlation characteristics of original and synthetic textures generated by STCPM and TCM, whose correlations are in a closest agreement to that of the original texture, the filter method is the next, while there is almost no temporal correlation in texture data generated by SCM. In Figure 11b, the spatial correlation of the texture generated by STCPM is also in closest agreement to that of the original texture, which is similar to the SCM, but the TCM is the worst. Although better than the TCM, the filter method performs a bigger deviation than the STCPM and SCM. As can be seen from Figure 11, the texture generated by STCPM has both temporal and spatial correlations, while the texture generated by TCM or SCM has only one correlation in the pulse dimension or range dimension. Besides, the STCPM has a closer agreement in both temporal and spatial correlations than the filter method. The difference between the data generated by STCPM and original data can be explained by the usage of the nonlinear transformation procedure for the reconstruction of texture.

**Figure 12:**
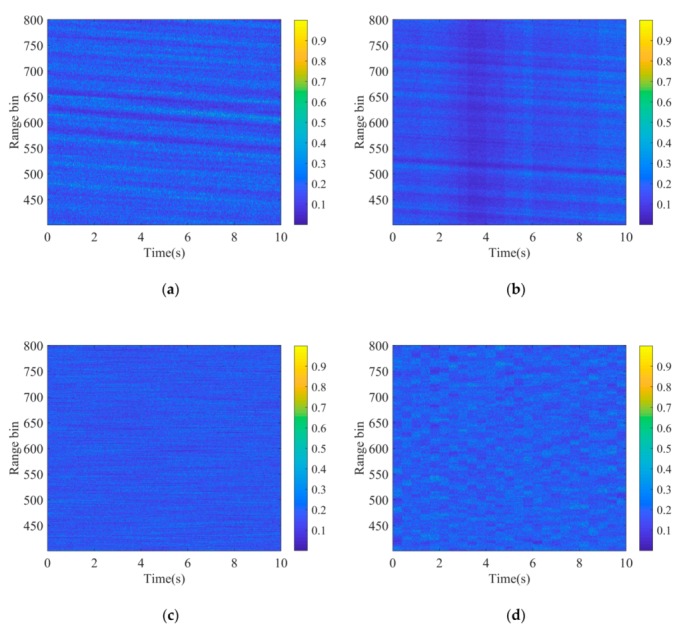
The amplitude-range-time of real and generated clutter: (**a**) Original; (**b**) STCPM; (**c**) TCM; (**d**) SCM.

Figure 12 presents the comparison of the normalized amplitude returns between the original and reconstructed sea clutter. The data generated by STCPM presents multiple oblique stripes similar to the original data, perhaps as a result of wind gusting, whereas the influence of wind is not considered in TCM and SCM. The vertical stripes appear in Figure 12b because the texture is assumed to be a constant in the coherence time. The fine stripes shown in 12c occur mainly because there is no correlation between the returns from different range bins. Similarly, the trivial data blocks in 12d are caused by the temporally correlated speckle and the spatially correlated texture. As can be seen, the clutter generated by STCPM fits the original data best in amplitude characteristic.

**Figure 13:**
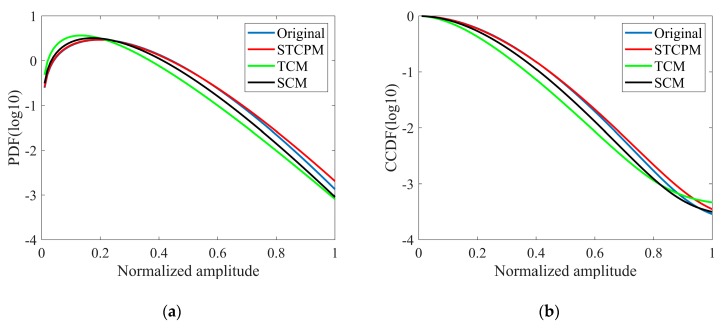
K-distribution fitting of real and generated clutter: (**a**) PDF; (**b**) CDF.

The comparison of the PDF and CDF between the original clutter and reconstructed clutter is shown in Figure 13. It can be seen that the clutter generated by STCPM is the most consistent with the original data. In order to quantify the performance of three methods, two test statistics—the root mean squared error (RMSE) and Kolmogorov–Smirnov (KS)—are calculated in this paper. As shown in Table 3, the two statistics for STCPM are the smallest of the three methods, which fits the original data best. The number of independent samples is much less because of the temporal correlation, which can explain the deviation in the tail of K distribution.

**Figure 14:**
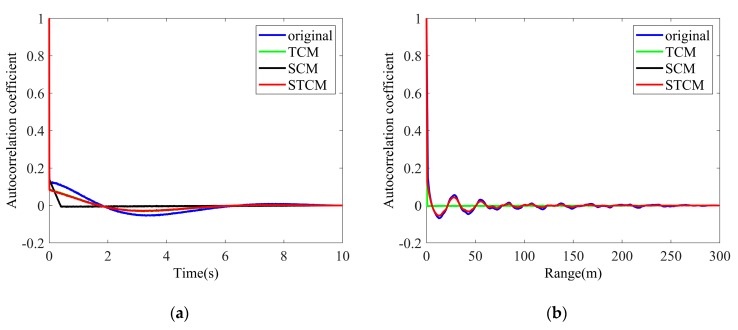
Average correlation coefficients of clutter amplitude: (**a**) Temporal correlation; (**b**) Spatial correlation.

Figure 14 presents the comparison of the correlation characteristics between the original sea clutter amplitude and reconstructed sea clutter amplitude. Similar to the performance in the reconstructed texture, STCPM takes the lead, which generates the clutter with desired temporal and spatial correlations, while TCM and SCM do not. There is also small difference between the original and reconstructed clutter, perhaps as the result of the nonlinear transformation in the texture generation and very small spatial correlation in the speckle component, which is assumed to be zero.

**Figure 15:**
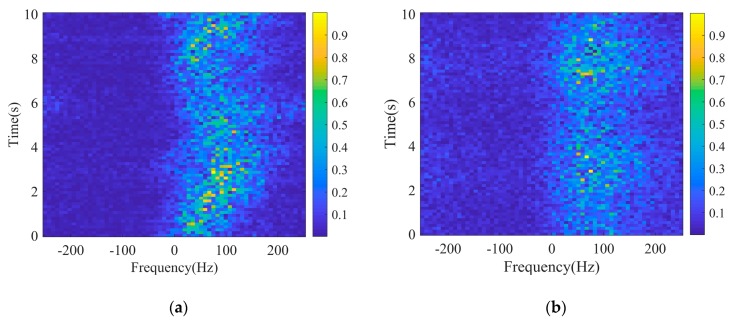
Doppler spectra of real and generated clutter (range bin = 300): (**a**) Original; (**b**) STCPM.

Figure 15 gives the results of the normalized evolving Doppler spectrum along time of the original clutter and reconstructed clutter generated by STCPM. Each spectrum was derived from 64 samples, equivalent to a time period of 128ms. It is clear that the spectrum of the original clutter appears asymmetric in shape, with a nonzero mean Doppler shift. Although the radar is fixed and there is no moving target on the sea, the sea surface fluctuates with a low velocity due to some natural factors such as the wind, which accounts for the Doppler shift. According to signal processing theory, the Doppler characteristics are relevant to the short-term correlation of clutter. Consequently, the Doppler characteristics of the clutter are mainly dependent on the speckle component. Actually, the Doppler spectrum changes over both time and range due to the nonstationary properties and the continuous fluctuations of the surface. In this paper, the method concentrates on only the temporal correlation of the speckle, without any spatial correlation of it. Thus, the variation of Doppler spectrum over range is not our focus here. There is no significant difference in the three generation methods of speckle, and as a result, only the result of the STCPM is presented here. As can be seen, there is an approximately equivalent mean Doppler shift in synthetic clutter, and although there is derivation in the variation of Doppler over time, the possible reason is the variation of short-term correlation of the speckle due to the non-stationarity of clutter, which is not considered in the paper. 

As can be seen from the above results, the proposed method can not only reproduce similar amplitude characteristics with that of the original clutter, but generate clutter with both temporal–spatial correlation, which cannot be achieved by the SCM and TCM. Compared with the filter method, the texture generated by STCPM is in a closer agreement to that of the original clutter in both temporal and spatial correlations, which demonstrates that the proposed method can reproduce the correlation characteristics of clutter more accurately.

## 6. Conclusions

A novel method has been proposed for simulating K-distributed sea clutter based on the correlation characteristics obtained from the real data. In the paper, the spatial–temporal correlated texture component is generated by the proportional reconstruction method and the temporally correlated speckle component is generated by the matrix transformation. An important result is that the method not only generates clutter data with both temporal and spatial correlations, but provides a more accurate reproduction of correlation characteristics compared with previous methods. In addition, the cross-correlation of speckle and coherence of texture are also considered in the reconstruction. Simulation results demonstrate that the proposed method is able to generate 2D K-distributed sea clutter returns with the specified correlation characteristics, and the evolving Doppler spectra along time is also similar to that of the original data. Further work may extend the method to the returns from a longer processing interval and more range bins, where the segmented reconstruction can be considered.

## Figures and Tables

**Table 1:** Characteristic parameters of radar.

Characteristic Parameter	Value
bandwidth	100 MHz
PRF	500 Hz
sample frequency	200 MHz
polarization	Vertical
antenna beam width	3 deg
grazing angle	8 deg

**Table 2:** Estimated parameters of amplitude distributions.

Distribution	Parameter	Value
Rayleigh	*v*_Rayl	0.1782
K	*v*_K	5.3078
	*c*_K	0.0540
Weibull	*v*_Wbl	1.8159
	*c*_Wbl	0.2461
Lognormal	*v*_Logn	−1.7120
	*c*_Logn	0.6766

**Table 3:** Statistics of methods.

Method	RMSE	KS
STCPM	3.9908 × 10^−6^	0.0081
TCM	0.0013	0.1746
SCM	1.7702 × 10^−4^	0.0678
